# Novel *PTCH1* mutations in Japanese familial nevoid basal cell carcinoma syndrome

**DOI:** 10.1038/s41439-020-00126-6

**Published:** 2020-11-18

**Authors:** Yoji Nakase, Atsuko Hamada, Naoya Kitamura, Tsuyoshi Hata, Shigeaki Toratani, Tetsuya Yamamoto, Tetsuji Okamoto

**Affiliations:** 1 Oral and Maxillofacial Surgery, Hiroshima University Hospital, Hiroshima, Japan; 2 Department of Oral and Maxillofacial Surgery, Kochi Medical School, Kochi University, Kochi, Japan; 3 Department of Oral Surgery, Kawasaki Medical School, Okayama, Japan; 4 Department of Molecular Oral Medicine and Maxillofacial Surgery, Graduate School of Biomedical and Health Science, Hiroshima University, Hiroshima, Japan; 5 Present Address: Kondo Dental Clinic, Medical Corporation Mutsumikai, Okayama, Japan

**Keywords:** Mutation, Diseases

## Abstract

Nevoid basal cell carcinoma syndrome (NBCCS), also known as Gorlin syndrome, is inherited in an autosomal dominant manner and is characterized by a combination of developmental abnormalities and a predisposition to tumor formation. Hedgehog receptor Patched 1 (*PTCH1*) has been identified as the mutated gene in NBCCS. We identified the PTCH1_c.3298_3299insAAG_p.1099_1100insE mutation in the transmembrane region, which comprises a sterol transporter whose abnormal function is reportedly related to pathogenicity.

Nevoid basal cell carcinoma syndrome (NBCCS, OMIM: 109400), also known as Gorlin syndrome, was first reported by Gorlin and Goltz in 1960^1^. NBCCS is an autosomal dominant inherited disease characterized by bifid ribs and palmar pits, as well as a predisposition to various tumors, including basal cell carcinoma (BCC), medulloblastoma, ovarioma, cardiac fibroma, odontogenic keratocyst, and skin patch^2–4^. At birth, patients with NBCCS typically exhibit macrocephaly or rib anomalies. As the patients' age, palmar and plantar pits become evident. At the age of approximately 10 years, a odontogenic keratocyst, formerly known as a jaw cyst, is observed as the first notable symptom in the diagnosis of NBCCS. At the age of approximately 20 years, BCC can develop at any location on the body, particularly on the eyelid. However, in Japanese patients with NBCCS, the incidence of BCC is significantly low, whereas the frequency of odontogenic keratocysts is relatively high^3,5^.

The gene responsible for causing NBCCS is the human homologue of the *Drosophila* patched gene Patched 1 (*PTCH1*)^6^. PTCH1, a Hedgehog (HH) receptor, is located on chromosome 9q22.3, consists of 23 exons, and encodes a 1447-amino-acid integral membrane protein with 12 transmembrane (TM) regions, five of which form a sterol-sensing domain and two extracellular loops at which the N-terminal domain of the HH ligand binds^7,8^. The PTCH1 protein functions to inhibit the transmembrane protein Smoothened (SMO). Once extracellular HH ligands bind to the PTCH1 receptor, PTCH1 releases SMO inhibition, allowing SMO to participate in downstream signaling and activate GLI transcription factors. HH signaling plays an essential role during embryogenesis and maintains stem cell populations in certain adult tissues^9,10^. Unliganded PTCH1 inhibits HH signaling; this repression is released when HH ligands bind to PTCH1^7,8^. In this study, we investigated *PTCH1* germline mutations in Japanese familial NBCCS.

Patients were diagnosed with NBCCS in the Department of Oral and Maxillofacial Surgery, Kawasaki Medical University Hospital, and Hiroshima University Hospital based on both clinical and genetic analyses. Each patient in the family with NBCCS was designated F1, F2, F3, F4, F5, F6, and F7. The pedigree is shown in Fig. 1a. F1–F5 had odontogenic keratocysts. F4 and F5 had undergone surgery for removal of odontogenic keratocysts several times at the Kawasaki Medical University Hospital. F1–F7 exhibited palmar and plantar pits, F3 and F4 showed kyphoscoliosis and calcification of the falx cerebri, and F4 had BCC, skin patch, and hairy skin patch. F6 and F7 had a congenital deficiency of the second premolar. Based on interviews with the patients, the husband of F5 also had a deficiency of the second premolar, and his mother had congenital deficiency of several incisors. The main phenotypes of F1–F7 are shown in Fig. 1b.

**Fig. 1:**
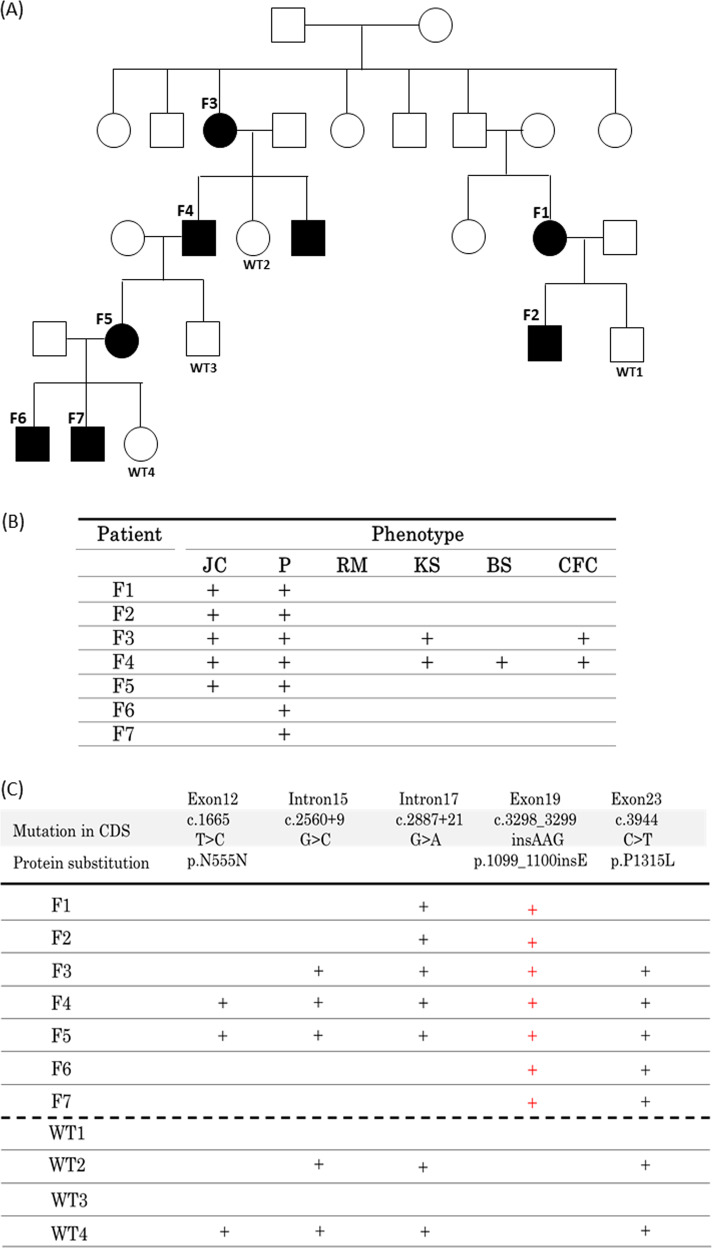
Pedigree, phenotype, and genotype of familial nevoid basal cell carcinoma syndrome (NBCCS). **a** Pedigree of familial NBCCS: NBCCS was inherited in four generations. **b** Phenotypes of familial NBCCS. JC: Jaw Cysts, P: Pits, RM: Rib Malformation, KS: Kyphoscoliosis, BS: Bridging of Sella, CFC: Calcification of Falx Cerebri (**c**) Genotypes of familial NBCCS.

We detected irregular band shifts in exon 19 in F1–F5 through PCR-SSCP (data not shown). Direct sequencing revealed the c.3298_3299insAAG mutation in exon 19 of *PTCH1* in F1–F7 (Fig. 2). To screen for other pathogenic mutations affecting the HH signaling pathway, we performed NGS with the MiSeq sequencer using the TruSight One panel for familial NBCCS and identified the following mutations: *PTCH1*_c.1665T>C_p.N555N (exon 12), _c.2560+9G>C (intron 15), _c.2887+21G>A (intron 17), _c.3298_3299insAAG_p.1099_1100insE (exon 19), and _c.3944C>T_p.P1315L (exon 23). Among the detected mutations, only PTCH1_c.3298_3299insAAG_p.1099_1100insE was specifically shared in our cases of familial NBCCS (F1–F7) and was not shared in WT1–WT4 (Fig. 1c). In contrast, there were no other pathogenic mutations in HH signaling-related molecules, such as *PTCH2*, *SHH*, *SMO*, *SUFU*, *GLI1*, *GLI2*, and *GLI3*.

**Fig. 2:**
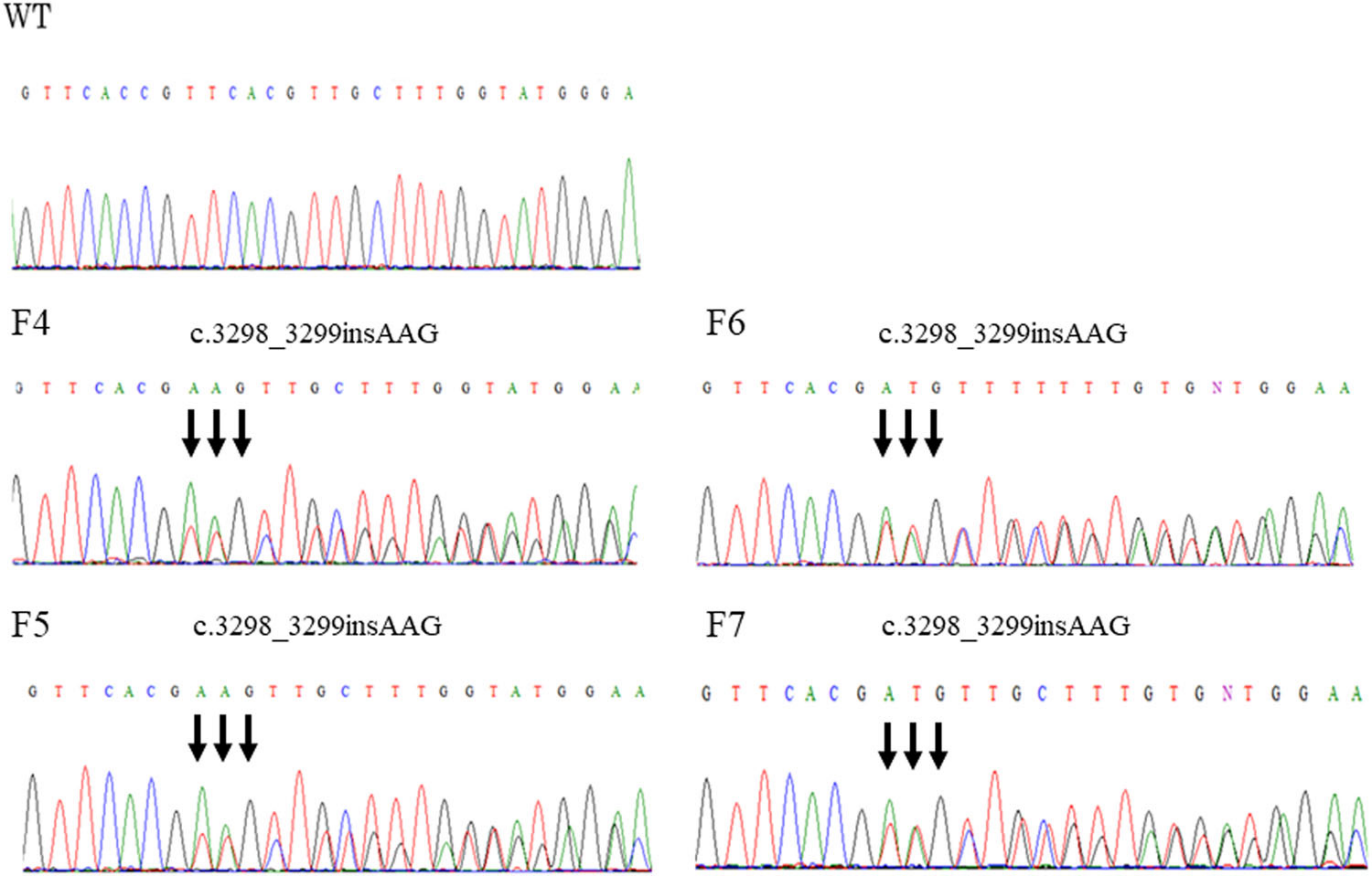
Direct sequencing of *PTCH1* in familial nevoid basal cell carcinoma syndrome (NBCCS). The results of direct sequencing of *PTCH1* exon 19 are shown. Though an insertion was not detected in WT, an AAG insertion was detected between coding sequences 3298 and 3299 in F4–F7.

The detected mutation, PTCH1_c.3298_3299insAAG_p.1099_1100insE, was located in the 10^th^ transmembrane region and mapped closely to sterol-binding sites. Recently, through structure-guided mutational analysis, Gong *et al*. revealed that the interaction between Shh-N and PTCH1 is steroid-dependent^11^. Moreover, *PTCH1*_c.3298_3299insAAG was not found in Togo Var, Exome Variant Server, dbSNP, dbVar, or ClinVar, confirming that the mutation is novel. In addition, *PTCH1*_c.3298_3299insAAG was predicted as disease causing by Mutation Taster2 and as deleterious by PROVEAN software. Thus, we predicted that the specifically shared mutation, PTCH1_c.3298_3299insAAG_p.1099_1100insE, would be responsible for the pathogenesis of NBCCS^11,12^.

Of note, c.1665T>C (rs1805155), located in a sterol-sensing domain, is synonymous and the most common SNP in *PTCH1*. Clinical significance is benign in ClinVar. To our knowledge, there is no prior report about the pathogenicity of this SNP. c.2560+9G>C and c.2887+21G>A are intronic SNPs. Although c.2887+21G>A is not reported, c.2560+9G>C (rs2066829) is registered as an intronic variant and benign in ClinVar. It is unknown whether the *PTCH1* polymorphisms located in introns cause a functional change. However, intronic polymorphisms have been demonstrated in association with other complex diseases, including the association of IRF6 with cleft lip/palate^13,14^. c.3944C>T (rs357564) results in an amino acid change in the topological domain of the C-terminus of PTCH1. Though the interpretation is benign in ClinVar, some researchers have concluded that, in combination with oral contraceptive use, c.3944C>T-carrier in *PTCH1* is associated with an increased risk of breast cancer^15^. Because PTCH1 has a sterol-sensing domain, long-term exogenous hormone use is reportedly related to breast cancer risk. Although the above three SNPs were also detected in wild-type *PTCH1* in this family, it was recently suggested that SNPs in key genes involved in the HH signaling pathway are associated with susceptibility to odontogenic cystic lesions^16^.

Here, we reported the detection of a novel mutation, PTCH1_c.3298_3299insAAG_p.1099_1100insE, in Japanese familial NBCCS. Therefore, we conclude that *PTCH1*_ c.3298_3299insAAG is the “likely pathogenic” mutation of NBCCS.

## Data Availability

The relevant data from this Data Report are hosted at the Human Genome Variation Database at 10.6084/m9.figshare.hgv.2927.
